# Localizing adult playfulness in dimensions of romantic attachment and jealousy in singles

**DOI:** 10.3389/fpsyg.2026.1776141

**Published:** 2026-04-07

**Authors:** Kay Brauer, René T. Proyer

**Affiliations:** Department of Psychology, Martin Luther University Halle-Wittenberg, Halle (Saale), Germany

**Keywords:** jealousy, playfulness, romantic attachment, romantic relationships, singlehood

## Abstract

Adult playfulness is a personality trait gaining increasing attention, particularly in romantic contexts. We localize four facets of playfulness (Other-directed, Lighthearted, Intellectual, and Whimsical) within dimensions of attachment styles and romantic jealousy. Earlier findings from couples showed that Other-directed playfulness relates to low avoidant attachment and all facets related to lower emotional expressions of jealousy. To expand the knowledge about the generalizability and overcome variance restrictions from couples’ data, we conceptually replicated earlier research using the same measures and analyzed data of *N* = 384 singles. As expected, singles showed greater insecure attachment than couples, whereas only cognitive jealousy was elevated in comparison to partnered individuals. Our findings replicated associations between playfulness and attachment, with Other-directed playfulness being the strongest predictor of lower avoidant attachment and Lighthearted playfulness showing a small negative association with anxious attachment. However, we found negligible associations with jealousy. We discuss the role of playfulness in singles and couples and whether the reframing process that characterizes playfulness might be less active in singles when it comes to jealousy in the sense of (re)interpreting actual and perceived relationship threats.

## 1 Introduction

Adult playfulness is a personality trait that describes individual differences in how people (re)frame situations in such a way that they experience them as personally interesting, and/or entertaining, and/or intellectually stimulating (Proyer, 2017). A recent structural model assumes four facets of playfulness [i.e., Other-directed, Lighthearted, Intellectual, and Whimsical (OLIW); Proyer, 2017], which account for differential findings regarding external variables. The role of playfulness in romantic relationships has gained strong interest (Brauer et al., 2021). In the present study, we extended the knowledge about playfulness in romantic life by addressing its associations with two constructs that are frequently studied as related but theoretically distinct descriptors of how people experience relationships, namely, romantic attachment styles (also referred to as attachment orientations) and jealousy. Brauer et al. (2024) previously tested the associations between playfulness, romantic attachment styles, and jealousy in couples, and we extended the knowledge about these associations by analyzing singles to learn more about the generalizability of the findings and the question of whether previously observed associations reflect dyad-specific processes (that could even be specific to opposite-sex relationships) or more general dispositional patterns, which should also be found in singles. Studying individuals that are not currently subject to reciprocal partner influences or relationship-specific effects may provide an even more direct assessment of the interplay of the variables under investigation or at least allow for an initial comparison of the coefficients.

The increasing number of singles in Western countries and the knowledge about differences in expressions of attachment, and to some degree jealousy, between singles and partnered people (Pepping et al., 2018) suggest the need to examine the replicability of findings from couples to generalize the knowledge regarding the localization of playfulness in multidimensional systems of attachment styles and jealousy from couples to singles. As mentioned earlier, studying a group in which current relationship dynamics and processes are absent, helps for a better understanding of what the real nature of the associations are when interpreted with respect to differences and similarities with earlier findings.

### 1.1 Playfulness and relationships

For decades, it has been assumed that trait-playfulness plays a role in how people form, experience, and maintain their relationships. For example, the “play face” is a facial expression that animals use to initiate non-threatening rough-and-tumble play, which can serve as a valuable tool for learning important survival skills and bonding (Bekoff and Allen, 1998). Although playfulness is comparatively understudied in human adults, several researchers have put forward hypotheses and findings about the role of playfulness in social relationships (for an overview, see Brauer et al., 2021). There is consensus that playful individuals can (re-)frame everyday situations in ways that allow them to express their love and bonding through novel and surprising behaviors (e.g., finding nicknames based on shared experiences), solve interpersonal conflict through their playfulness (e.g., making humorous remarks during serious discussions), and break routines, which contribute to maintaining relationships. Accordingly, it has been assumed that playfulness is a desired trait in partners: The *Signal Theory of Playfulness* asserts that playfulness signals underlying traits, such as non-aggressiveness in men and fecundity in women (Chick, 2001). This theory has received empirical support from studies which found that playfulness is a strongly desired trait in partners and young, middle-aged, and older-aged couples, indicating assortative mating preferences (Brauer et al., 2023, 2024; Chick et al., 2020; Proyer et al., 2019).

Initial findings showed that playfulness relates to positive emotions, satisfaction, and love styles when analyzing individuals (Aune and Wong, 2002; Proyer, 2014; Proyer et al., 2018), and there is robust evidence that playful couples experience stronger benefits from couple therapy as opposed to less playful couples (Metz and Lutz, 1990). Actor-Partner Interdependence Model analyses of couples showed that playfulness is positively connected to relationship satisfaction, particularly regarding Other-directed and Intellectual playfulness (Brauer et al., 2023; Proyer et al., 2019). However, there might be associations with negatively valued phenomena. For example, partners of those with higher expressions of Lighthearted (and to some degree Whimsical) playfulness reported greater mistrust concerning their partner. Hence, while most studies noted the contribution of playfulness to indicators of relationship quality, there are aspects that elicit potential negative consequences that require further exploration.

#### 1.1.1 Studying associations with attachment styles and romantic jealousy

To better understand the potential mechanisms that contribute to the associations between playfulness and relationship satisfaction, Brauer et al. (2024) tested the associations between the four facets of playfulness with indicators of romantic attachment and jealousy. Attachment styles are interindividual differences in working models of relationships that contain expectations about reactions and interactions with one’s partner among two orthogonal dimensions: *anxiety* (i.e., worries about the relationship and the partner’s reliability) and *avoidance* (i.e., dismissing close relationships and avoiding closeness; Hazan and Shaver, 1987). The past 15 years have seen a shift in the understanding of adult attachment styles, as they are more malleable than initially presumed, with longitudinal studies challenging the notion that attachment in young age determines adult attachment and showing that relationship experiences update the “inner working models” of relationships (e.g., Chopik et al., 2024; Fraley, 2019). For example, secure people can become more insecurely attached after their trust was violated (e.g., after being cheated on) and insecurely attached people can become more secure when experiencing that their partner provides a “safe haven” as attachment theorists describe partners, who are reliable and provide trust and love to their significant other. Also, there is evidence that attachment styles differ with regard to attachment figures, as one can hold different attachment orientations to parents, friends, and partners (e.g., Brumbaugh and Fraley, 2007; Fraley et al., 2011). Brauer et al. (2024) argued that trait playfulness might contribute to understand the complexity of how people experience and engage in their relationships and how this translates to attachment styles.

Romantic jealousy describes how people react to actual and perceived threats to their relationship, and can be expressed on *cognitive* (e.g., thinking that the partner intends to leave the relationship), *emotional* (e.g., feeling upset when the partner mentions another person), and *behavioral* levels (e.g., searching through the belongings of one’s partner; Pfeiffer and Wong, 1989). Romantic attachment and jealousy are interrelated but yet distinct, as data from a German sample comprising 656 participants show: While all types of jealousy relate to the anxious attachment style positively with moderate effect sizes, only cognitive jealousy relates to the avoidant attachment style, whereas emotional and behavioral types of jealousy are unrelated (Brauer and Proyer, 2025b). Both attachment and jealousy independently predict numerous relationship outcomes such as relationship satisfaction and relationship status (Attridge, 2013; Brauer et al., 2020; Shaver and Brennan, 1992; Simpson, 1990). Accordingly, findings on playfulness in couples have been extended to these variables.

To examine how the OLIW facets relate to attachment and jealousy, Brauer et al. (2024) analyzed data from 332 mixed-gender and 139 same-gender couples with APIMs. First, they found the associations between playfulness, jealousy, and attachment were invariant between mixed- and same-gender couples. Second, only Other-directed playfulness had a negative relationship with the avoidant attachment style, whereas those higher in Lighthearted and Intellectual playfulness showed minor negative associations with the anxious attachment style. Hence, in couples, playfulness showed only a few associations with attachment styles. Regarding jealousy, all facets of playfulness related to lower emotional jealousy, but those higher in Whimsical showed greater inclinations to cognitive and behavioral expressions of jealousy.^1^ These findings met the authors’ expectations and showed that playfulness (particularly Other-directed) corresponds with minor inclinations to secure attachment (i.e., low avoidant and anxious attachment styles) and less emotional jealousy (except for Whimsical playfulness).

### 1.2 The present study

The generalizability of available findings on attachment and jealousy in relation to playfulness is limited to couples (Brauer et al., 2024) but knowledge about the generalizability to singles is desirable (Park et al., 2024). For example, there is robust evidence that those in relationships show, on average, a more secure attachment style and more jealousy than singles (Brauer et al., 2020; Brauer and Proyer, 2020; Pepping et al., 2018; Valentova et al., 2020), but effect sizes of the differences between samples of singles and partnered persons are of small-to-moderate size. However, considering the variance restriction and the elevated mean levels when comparing singles and partnered individuals concerning attachment and jealousy, as well as the increasing evidence that singlehood is to some degree a function of attachment styles (for a discussion, see Pepping et al., 2018), it is important to extend the study of playfulness in relation to attachment and jealousy to the population of singles to overcome the methodological limitations from relying on couples. Therefore, we collected data from singles and conducted a replication study by using the same measures as Brauer et al. (2024), which allows to provide initial knowledge about the comparability between findings from singles and couples (Park et al., 2024).

In line with earlier research (Brauer et al., 2024; Proyer, 2014), theoretical notions that playfulness helps in establishing relationships (see Brauer et al., 2021; Chick, 2001), and the knowledge about the malleability of attachment models based on relationship experiences (e.g., Chopik et al., 2024), we examined the associations between four facets of playfulness and attachment and expected that playfulness is negatively associated with insecure attachment. On the facet level, we expected that Other-directed playfulness would correlate with lower avoidance and anxiety, and Lighthearted and Intellectual playfulness would correlate with low anxiety. Regarding jealousy, we examined its relationship with playfulness in exploratory fashion. While data collected from couples (Brauer et al., 2024) could help in replication, the reframing process that characterizes playfulness might be less important for dealing with relationship threats in singles because of the lack of an actual partner and relationship to worry about.

## 2 Methods

### 2.1 Sample and procedure

Our sample included 384 participants (258 women, 119 men, five non-binary, and two did not disclose gender). Our participants were between 18 and 60 years of age (*M* = 24.6, SD = 6.1). All participants reported not being in a romantic relationship at the time of the study. Most participants were students (83.1%), 14.1% were working professionals, 1.0% were in vocational training, 1.0% provided a voluntary social service, and 0.5% were unemployed.

We advertised our study as research on personality and romantic life during singlehood through flyers on-campus and online at our department’s website and social media. We asked participants to complete our online questionnaire (hosted by soscisurvey^2^) if they were ≥18 years of age, fluent in German, and currently not in a romantic relationship. Data collection took place between August 2023 and April 2024. Participants completed the questionnaire in about 15 min. We did not financially compensate participants, but psychology students could earn course credit. We only downloaded complete data sets (i.e., no missing values).

### 2.2 Instruments

We used the OLIW questionnaire (Proyer, 2017) to assess adult playfulness. It contains 28 items that assess the facets Other-directed, Lighthearted, Intellectual, and Whimsical, with seven items each (e.g., Other-directed: “I use my playfulness to cheer others up”). Participants give their responses on a seven-point rating scale (1 = *strongly disagree*, 7 = *strongly agree*). There is robust evidence for its validity, for example, regarding structural validity (e.g., confirmatory factor analyses), predictive validity (e.g., overlap with diary data of playfulness aggregated across 14 days), and nomological validity (e.g., correlations with theoretically near constructs), according to prior research (Proyer, 2017). The internal consistencies were in the expected range for short scales (Ziegler et al., 2014), with coefficients between 0.60 and 0.80, and retest correlations were high over intervals up to 3 months (≥ 0.67).

We assessed attachment styles with the Experiences in Close Relationships (ECR) questionnaire [Brennan et al., 1998; German version by Neumann et al. (2007)]. The ECR assesses attachment anxiety (e.g., “I worry that romantic partners won’t care about me as much as I care about them”) and avoidance (e.g., “I don’t feel comfortable opening up to romantic partners”), with 18 items each. Note that the instruction of the ECR states that it is not the aim “to record the current status of a particular relationship, but how you feel and behave in your relationships in general.” Participants are asked how they generally experience relationships and indicate their agreement to the items on a seven-point rating scale (1 = *completely disagree*, 7 = *completely agree*). There is robust evidence of the good psychometric properties and validity of the ECR-R in its original and German versions (αs ≥ 0.85; Neumann et al., 2007), including strong measurement invariance between singles and couples (Brauer and Proyer, 2025a).

We used the German version of Pfeiffer and Wong’s (1989) Multidimensional Jealousy Scale (MJS) that includes scales for cognitive, emotional, and behavioral jealousy, with eight items each. Example items are “I think my partner is secretly developing a relationship with someone of the opposite sex” (Cognitive) and “I question my partner about his or her whereabouts” (Behavioral), and participants are asked to describe their experiences in relationships in general and rate these statements on a seven-point scale (1 = *never*, 7 = *always*). The items of the Emotional scale (e.g., “Your partner is flirting with someone of the opposite sex”) are evaluated with the scale anchors 1 = *very pleased* and 7 = *very upset*. As with the ECR, participants are asked to rate their experiences in general and not regarding a specific relationship. Pfeiffer and Wong (1989) reported good internal consistencies (≥0.85), a robust three-factor solution, and nomological validity across three samples. These findings have been replicated and extended for the German-language version, showing measurement invariance between genders, nomological and predictive validity, and temporal stability when testing couples across 5–9-month intervals (≥0.60; Brauer and Proyer, 2025b).

### 2.3 Data analysis

For preliminary analyses, we compared the data from our samples of singles with those of couples, using Hedges’ *g* effect size (*g* = 0.20/0.50/0.80 indicating small/medium/large effects). Further, we computed bivariate Pearson correlations analyses between the measures of playfulness and attachment and jealousy along with bootstrapped 95% confidence intervals (CIs) based on 5,000 random samples. We assumed statistical significance when the CI did not contain zero and interpreted the effect sizes as small, medium, and large when *r*s ≥ 0.10, 0.20, and 0.30 (Funder and Ozer, 2019). Power analyses (type = sensitivity) with G*Power (Faul et al., 2009) showed that our sample size allowed us to detect small-to-medium effect sizes (*r*s ≥ 0.15) with 90% power and 5% type I error rate. The sample size meets requirements for estimating stable correlation parameters (Schönbrodt and Perugini, 2013).

## 3 Results

### 3.1 Preliminary analyses

Table 1 shows that all measures yielded comparable internal consistencies to prior research; coefficients were between 0.68 and 0.92. The OLIW questionnaire scores were comparable to prior research that tested individuals and couples, indicating no robust differences in comparison to couples (Brauer et al., 2024; Proyer, 2017). As expected, our sample showed, on average, greater expressions of avoidance and anxiety and more variability than couples have. Compared to the couple data by Brauer et al. (2024), our singles showed differences regarding attachment styles, with a large effect size for avoidance (Hedges’ *g* = 1.20) and a medium effect size for anxiety (*g* = 0.59). For jealousy, we found elevated expressions only for cognitive jealousy (*g* = 0.83; *g*s = 0.06 and 0.19 for emotional and behavioral jealousy). All study variables showed negligible associations with age and gender.

**TABLE 1:** Descriptive statistics and internal consistencies of study measures.

Variable	*M*	SD	α
Playfulness			
Other-directed	4.94	0.93	0.71
Lighthearted	4.00	1.04	0.79
Intellectual	4.09	0.89	0.68
Whimsical	4.16	1.07	0.81
Attachment			
Anxiety	3.91	0.99	0.91
Avoidance	3.33	0.97	0.92
Jealousy			
Cognitive	3.04	1.27	0.91
Emotional	4.38	0.95	0.86
Behavior	2.19	0.89	0.85

*N* = 384.

### 3.2 Playfulness and attachment styles

As in couples, Other-directed playfulness showed a robustly negative association with the avoidant attachment style, but contrary to the findings from couples, those higher in Lighthearted and Intellectual playfulness showed also lower expressions in the avoidant attachment style (*r*s = −0.18 and −0.22, *p*s < 0.001; see Table 2). Next, we computed a regression analysis with the OLIW facets as predictors of attachment avoidance. After controlling for the shared variance, only Other-directed playfulness remained a robust predictor (β = −0.39, *b* = −0.41, *p* < 0.001), whereas the associations of the remaining facets with the avoidant attachment style were negligible (all βs and *b*s < 0.05, *p*s ≥ 0.357). This mirrored the findings from couples well, but the association with Other-directed was numerically stronger compared to Brauer et al.’s (2024) findings from couples (*b* = −0.14). As in the couples, we found Lighthearted playfulness had a negative relationship with the anxious attachment style (*r* = −0.18, *p* < 0.001).

**TABLE 2:** Correlations between playfulness and romantic attachment and jealousy.

	Attachment styles		Jealousy		
	Avoidance	Anxiety	Cognitive	Emotional	Behavioral
Other-directed	−0.41***	0.04	−0.10	−0.04	−0.02
	[−0.50, −0.33]	[−0.07, 0.15]	[−0.20, 0.01]	[−0.15, 0.06]	[−0.13, 0.10]
Lighthearted	−0.18***	−0.18***	−0.12*	−0.11*	−0.08
	[−0.29, −0.08]	[−0.28, −0.08]	[−0.21, −0.02]	[−0.19, −0.02]	[−0.17, 0.02]
Intellectual	−0.22***	−0.09	−0.10	−0.12*	−0.11*
	[−0.33, −0.11]	[−0.20, 0.02]	[−0.21, 0.01]	[−0.22, −0.03]	[−0.20, −0.01]
Whimsical	−0.10*	−0.03	0.04	0.03	0.02
	[−0.22, 0.01]	[−0.13, 0.08]	[−0.08, 0.14]	[−0.07, 0.13]	[−0.08, 0.13]

*N* = 384.

*p < 0.05.

***p < 0.001. Two-tailed. *P*-values computed with bootstrapped standard errors (*k* = 5,000 random samples). Bootstrapped 95% confidence intervals in brackets.

### 3.3 Playfulness and romantic jealousy

As in couples, there was a minor effect size that described the association of cognitive jealousy with Lighthearted playfulness (*r* = −0.12, *p* = 0.020; see Table 2). Contrary to Brauer et al.’s (2024) findings from couples, where all facets of playfulness have related negatively to emotional jealousy, we found only minor effect sizes for Lighthearted (*r* = −0.11, *p* = 0.032) and Intellectual (*r* = −0.12, *p* = 0.016) playfulness. Finally, in line with data from couples, behavioral expressions of jealousy were unrelated to playfulness, except for a minor effect size regarding Intellectual playfulness (*r* = −0.11) that should not be over-interpreted, considering the *p*-value of 0.041 and the upper limit of the 95% CI (−0.005), which is marginally different from zero.

## 4 Discussion

The present study added knowledge about the localization of playfulness in romantic life by analyzing the associations with romantic attachment styles and jealousy in singles. Since those in relationships show lower avoidant and anxious attachment styles and higher jealousy than singles (e.g., Brauer et al., 2020; Valentova et al., 2020), the replication of findings derived from couples with singles is needed to clarify the generalizability of past results. Using the same measures (i.e., multidimensional instruments to assess playfulness, attachment, and jealousy) as Brauer et al. (2024) allowed us to examine whether prior findings would translate to singles. In line with the literature, we found elevated expressions of avoidant and anxious attachment styles in our sample of singles, compared to Brauer et al.’s (2024) couples. Effect sizes were moderate to large. Regarding jealousy, only cognitive expressions in our sample were robustly different from couples. To our knowledge, this was the first comparison of jealousy between singles and couples using Pfeiffer and Wong’s (1989) three-dimensional model and instrument. Our findings show the importance of differentiating between the types of jealousy. Beyond the expected mean differences between samples, we were interested in whether associations between playfulness, attachment, and jealousy as found in couples would hold in singles.

### 4.1 Playfulness and attachment styles

The associations between the OLIW facets and attachment styles were comparable to Brauer et al.’s (2024) study of couples, with Other-directed playfulness being the best predictor of the avoidant attachment style and Lighthearted playfulness correlating with low expressions in the anxious attachment style. The latter replicated this finding from couples well and is in line with Brauer et al. (2024); it can be assumed that the lighthearted outlook on approaching life and preferring to improvise when dealing with stressful events (Tandler et al., 2024) also translates to the domain of relationships, for example, when dealing with situational interpersonal rejection, and not assuming a dichotomous view (e.g., obsessive worrying about the partner ending the relationship).

While our findings are consistent with previous research on couples (Brauer et al., 2024), our analysis suggests that the relationship between Other-directed playfulness and the avoidant attachment style is numerically stronger among singles. Considering the large effect size in differences of expressions of the avoidant attachment style between couples and our sample, relationship status might contribute to understand the findings. From a methodological perspective, this is likely a consequence of the decrease of range restriction and greater variability in attachment expressions in our sample, contributing to greater covariance and correlation coefficients (Aguinis and Whitehead, 1997). From a theoretical perspective, our finding might highlight the role of playfulness in close relationships, showing that those enjoying using their playfulness to cheer others up, solve disagreements, and make relationships interesting (i.e., Other-directed playfulness) report less of an inclination to dismiss close relationships and avoid emotional closeness. At the same time, secure attachment provides the basis for trust, communication, and dyadic adjustment (e.g., Chopik et al., 2024), and it can be well-argued that securely attached people might be inclined to develop a more playful approach to their relationships. More knowledge on the co-development of these variables is desirable. Finally, we note that the differences between findings of singles and couples are likely more complex than can be explained by relationship status alone.

While our data are cross-sectional and do not contain information about the length of singlehood, it would be interesting to examine whether (Other-directed) playfulness can contribute to reducing the likelihood of remaining single over time. The decision to remain single is increasingly being examined through the lens of attachment theory, with findings suggesting that individuals with different attachment styles may have varying experiences of singlehood, ranging from contentment to distress (Pepping et al., 2025). Accordingly, one might examine whether those higher in Other-directed playfulness show a greater likelihood of finding a partner (if desired) and are open to entering a relationship, as the playful approach to relationship could ease dealing with close relationships. Initial findings from a large sample of 1,191 Brazilians showed that Other-directed playfulness was positively related to entering short- and long-term relationships (de Moraes et al., 2021), but they did not consider attachment styles in this study. The contribution of both playfulness and attachment concerning relationship formation (and being voluntarily single) should be studied in future research with a longitudinal design. Furthermore, we recommend learning more about the potential causal mechanisms and co-development of playfulness and attachment. The literature suggests that playful exploration of the social environment during childhood relates to developing a secure attachment in the sense of working models characterized by openness toward relationships and trust during youth and adulthood (Alcock, 2013; Gordon, 2014).

Finally, trainings might reveal more about the malleability and effects of playfulness on attachment styles, and vice versa. Proyer et al. (2021) conducted a placebo-controlled study to evaluate the effectiveness of three brief exercises designed to enhance playfulness (e.g., counting playful moments, recalling three playful experiences, and applying playfulness in a new context). The exercises were successful in increasing playfulness and were associated with improved well-being and reduced depressive symptoms, and the effects lasted at least 4 weeks post-training). Using this design would help examine potential changes in attachment. Also, Azani Sadka et al. (2024) recently proposed playful group interactions in the context of group psychotherapy to revise working models and enhance attachment security. They argued that playful encounters with others in a “secure” therapeutic environment allows insecurely attached people to engage with others as a byproduct, for example, when solving a game-based problem as a group, instead of being assigned social interaction as a homework (e.g., “speak with a stranger”). Azani Sadka et al. (2024) noted that initial evidence showed that this approach allows participants to update and guide their working models of relationships toward a more secure attachment style. Another avenue to learn more about the interplay between playfulness and attachment could be achieved by examining emotion-focused therapy (EFT; Greenberg and Elliott, 2002) that aims to help people identifying, processing, and dealing with emotions in a way that contributes to mental health. EFT takes principles of attachment theory into account, and one part of this training is reframing emotions in an adaptive manner and playfulness could contribute to this process. Rubinstein et al. (2025) provided evidence that the OLIW facets relate to positively to adaptive emotion strategies (*r*s between 0.14 and 0.35). Considering the fruitful implementation of playful elements in therapeutic domains, including successful trainings of playfulness, and the present and recent findings on playfulness and attachment, further research on their interplay should reveal more useful information about the role of playfulness in relationships.

### 4.2 Playfulness and jealousy

Contrary to Brauer et al. (2024), who reported from couples that all facets of playfulness related negatively to emotional jealousy, and Whimsical playfulness showed positive inclinations to cognitive and behavioral expressions of jealousy, our data from singles did not replicate these findings. While we found negative associations between Lighthearted and Intellectual playfulness with emotional jealousy, the associations were of minor effect sizes, and the remainder of the correlations were negligible, suggesting that playfulness is unrelated to expressions of jealousy in singles.

While there is evidence for the temporal stability of the MJS scores from individuals and couples (Brauer and Proyer, 2025a; Pfeiffer and Wong, 1989), and jealousy is assumed to be a trait-like variable, there is little knowledge about how romantic jealousy is expressed in singlehood and relationships, especially regarding Pfeiffer and Wong’s (1989) dimensions of jealousy. Valentova et al. (2020) found no differences in jealousy expressions between singles and partnered individuals when it came to anticipated sexual jealousy, but it is unclear whether general inclinations to romantic jealousy are similarly expressed when having an actual partner and a relationship that can be worried about, versus being single and thinking about a hypothetical partner. Also, it is possible that jealousy is differently distributed and expressed between the populations of singles and partnered individuals. Similarly to differences in attachment styles between the populations, it might be possible that partnered individuals show greater sensitivity to cues that might signal threats to their relationship, whereas romantic jealousy is comparatively inactive among singles. Again, the reframing process of playful people might be negligible in singles because there is a lack of threat and sensitivity to cues that signal threat. Research that tracks singles over time and during transitions into romantic relationships might address the co-development of playfulness and jealousy and determine whether the interpretations of threat-related cues is related to reframing processes and specific facets; for example, Other-directed playfulness may help trigger positive memories of shared experiences and reduce feelings of jealousy and jealous thinking. At the same time, as in couples, it is possible that such reframing might contribute to elevate jealousy, as intellectual components of playfulness might also contribute to increasing jealousy-related cognitions in similar ways that have been discussed in the field psychiatry. In short, it has been hypothesized that excessive reframing processes might help understand psychiatric disorders that involve dysregulation in thinking, such as generalized anxiety (see Berger et al., 2018).

### 4.3 Limitations and future directions

Our study had several limitations. First, generalizability is limited to German-speaking participants, and replication of our findings with samples from other countries is desirable. Second, we only considered self-reports, which introduce potential biases and overestimations of correlations (Campbell and Fiske, 1959). Third, we did not collect data on our participants’ duration of their singlehood, desire for entering a relationship, and satisfaction with singlehood. Considering Pepping et al.’s (2018, 2025) work on singlehood, it is increasingly evident that singlehood is a diverse phenomenon from a psychological perspective, and future research should distinguish between the motives, desires, and needs of singles and acknowledge the heterogeneity of this population. It would be valuable to examine how these variables affect our findings. Fourth, our data are cross-sectional and do not allow conclusions about causality or about the potential mechanisms or underlying processes that might account for the associations found in this research. However, comparing our data from singles with those from same- and mixed-gender couples was a first step in generalizing findings on the localization of playfulness in systems of romantic attachment and jealousy, but further understanding these differences through identifying moderator and mediator variables is desirable. For example, relationship-specific obsessive thoughts might play a role as mediators (Brauer and Borchardt, 2025). Finally, it might be argued that statistical tests comparing the associations between singles and couples would be desirable, but it must be noted that the data structure and analytic approaches between those from individuals (in our case singles) and couples (i.e., interdependence between predictor- and outcome variables and determination of outcome variables through actor- *and* partner effects) is fundamentally different and do not allow for meaningful statistical tests of invariance.

Our study contributes to the generalization of findings regarding playfulness in romantic life by learning more about expressions and correlates of playfulness, attachment, and jealousy in singles, a rarely studied population receiving increasing interest. We hope this research helps to inform potential applications such as trainings of playfulness (Proyer et al., 2021) and in evaluations of the use of playful elements to increase attachment security in group settings (Azani Sadka et al., 2024).

## Data Availability

The datasets presented in this study can be found in the Open Science Framework under https://osf.io/prjkm/.

## Footnotes

1 Here, we focus on within-person (actor) effects. There were also partner effects, showing that Lighthearted playfulness related to *partners’* cognitive jealousy, and partners of whimsically playful people reported lower emotional jealousy. This mirrors earlier studies which showed that partners of lighthearted and whimsically playful people reported mistrusting their partner (Brauer et al., 2023; Proyer et al., 2019).

2
www.soscisurvey.de
